# Involvement of *luxS* in Biofilm Formation by *Capnocytophaga ochracea*

**DOI:** 10.1371/journal.pone.0147114

**Published:** 2016-01-22

**Authors:** Kyoko Hosohama-Saito, Eitoyo Kokubu, Kazuko Okamoto-Shibayama, Daichi Kita, Akira Katakura, Kazuyuki Ishihara

**Affiliations:** 1 Department of Oral Medicine, Oral and Maxillofacial Surgery, Tokyo Dental College, 5-11-13 Sugano, Ichikawa, Chiba, Japan; 2 Department of Microbiology, Tokyo Dental College, 2-9-18 Misaki-cho, Chiyoda-ku, Tokyo, Japan; 3 Department of Periodontology, Tokyo Dental College, 2-9-18 Misaki-cho, Chiyoda-ku, Tokyo, Japan; Oregon Health & Science University, UNITED STATES

## Abstract

*Capnocytophaga ochracea* is present in the dental plaque biofilm of patients with periodontitis. Biofilm cells change their phenotype through quorum sensing in response to fluctuations in cell-population density. Quorum sensing is mediated by auto-inducers (AIs). AI-2 is involved in intercellular signaling, and production of its distant precursor is catalyzed by LuxS, an enzyme involved in the activated methyl cycle. Our aim was to clarify the role of LuxS in biofilm formation by *C*. *ochracea*. Two *luxS*-deficient mutants, TmAI2 and LKT7, were constructed from *C*. *ochracea* ATCC 27872 by homologous recombination. The mutants produced significantly less AI-2 than the wild type. The growth rates of these mutants were similar to that of the wild-type in both undiluted Tryptic soy broth and 0.5 × Tryptic soy broth. However, according to crystal violet staining, they produced significantly less biofilm than the wild type. Confocal laser scanning microscopy and scanning electron microscopy showed that the biofilm of the TmAI2 strain had a rougher structure than that of the wild type. Complementation of TmAI-2 with extrinsic AI-2 from the culture supernatant of wild-type strain did not restore biofilm formation by the TmAI2 strain, but complementation of LKT7 strain with *luxS* partially restored biofilm formation. These results indicate that LuxS is involved in biofilm formation by *C*. *ochracea*, and that the attenuation of biofilm formation by the mutants is likely caused by a defect in the activated methyl cycle rather than by a loss of AI-2.

## Introduction

Dental biofilm is a dynamic microbial community composed of huge numbers of microorganisms whose profile changes in infectious disease such as periodontitis and dental caries [1]. *Capnocytophaga* are CO_2_-dependent Gram-negative rods with a gliding motility, and are found in subgingival dental plaque [2]. Increased prevalence of Capnocytophaga species including *Capnocytophaga ochracea* has been reported in several cases of periodontal disease [3–5]. *C*. *ochracea* has been frequently detected in elevated numbers within epithelial cells of site with periodontally diseased sites [6]. *C*. *ochracea* causes opportunistic infections such as sepsis and brain abscesses in immunocompromised patients [7, 8]. Recently, *C*. *ochracea* was isolated from noma lesions [9]. Capnocytophaga species in the oral cavity were reported to be associated with low birth-weight [10] and increased prevalence of Capnocytophaga species has been reported in patients with diabetes [11, 12].

Bacterial cells in biofilms communicate with each other in response to fluctuations in cell-population density and regulate their gene expression in a system called quorum sensing [13]. The communication is mediated by chemical signal molecules called auto-inducers (AIs) [14]. Auto-inducer-2 (AI-2) is derived from 4,5-dihydroxy-2,3-pentanedione, which in turn is derived from *S*-ribosylhomocysteine through the action of the enzyme LuxS [15]. LuxS, which is encoded by the *luxS* gene, occurs in a wide variety of Gram-negative and Gram-positive bacteria and is involved in intra- and inter-species cell communication [16]. Mutualistic and abundant biofilm growth of *Actinomyces naeslundii* T14V and *Streptococcus oralis* 34 in flowing saliva depends on the production of AI-2 by *S*. *oralis* 34 [17]. In the oral cavity, colonization by *C*. *ochracea* occurs in the early stage of dental plaque formation [18]. We previously reported that *C*. *ochracea* produces AI-2, which enhances colonization by *Fusobacterium nucleatum* [19], which then acts as a “bridge” between early and late colonizers in dental plaque. However, the role of *C*. *ochracea* in intra- and inter-species communication in biofilm formation has not been clarified. Here, we investigated the role of LuxS in biofilm formation by *C*. *ochracea*.

## Materials and Methods

### Bacterial strains and culture conditions

The bacterial strains used in this study are listed in Table 1.

**Table 1:** Bacterial strains and plasmids.

Strain or plasmid	Description	Source or reference
**Strain**		
*Capnocytophaga ochracea* ATCC 27872	wild type	American Type Culture Collection
*Capnocytophaga ochracea* TmAI2	*luxS*∷*ermFermAM*	This study
*Capnocytophaga ochrachea* LKT7	*luxS*∷*tetQ*	This study
*Capnocytophaga ochracea* luxS-C3	*tet*∷*ermF-luxS*	This study
*Escherichia coli* DH5α		Invitrogen
*Vibrio harveyi* BB170	*luxN*∷Tn5, AI-1 sensor**-**, AI-2 sensor**+**, reporter strain	[38]
**Plasmid**		
pCR2.1-TOPO	Km^r^, Ap^r^	Invitrogen
pVA2198	Em^r^	[39]
pKD375	Tet^r^, Ap^r^	[40]
pAI2	Km^r^, Ap^r^, Em^r^	This study
pAITQ	Km^r^, Ap^r^, tetQ^r^	This study
pLC2	Km^r^, Ap^r^, Em^r^	This study

*Capnocytophaga ochracea* ATCC 27872 (wild type) was maintained on a blood agar plates (BAPs) prepared from Tryptic soy (TS) agar (Becton Dickinson, Sparks, MD, USA) supplemented with hemin (5 μg/ml), menadione (0.5 μg/ml), and 10% defibrinated horse blood (Nippon Bio-Test Laboratories Inc., Tokyo, Japan) at 37°C under anaerobic conditions (N_2_, 80%; H_2_, 10%; CO_2_, 10%) in an anaerobic chamber (ANX-3, Hirasawa, Tokyo, Japan). The *C*. *ochracea* TmAI2 and luxS-C3 were maintained on a BAPs containing 10 μg/ml erythromycin (Sigma-Aldrich, St. Louis, MO, USA), and *C*. *ochracea* LKT7 was maintained on a BAPs containing 1.5 μg/ml tetracycline (Sigma-Aldrich). To investigate biofilm formation, *C*. *ochracea* was grown in TS broth supplemented with hemin (5 μg/ml) and menadione (0.5 μg/ml). TS broth with 1/2 dilution (i.e., 0.5 × TS broth) was also used, since biofilm formation is reported to be increased under suboptimal nutrient concentrations [20]. The growth of *C*. *ochracea* was determined by absorbance at 660 nm by using a spectrophotometer (UV-2550, Shimadzu, Tokyo, Japan). *Escherichia coli* DH5α was grown aerobically at 37°C on Luria-Bertani (LB) agar plates (Wako Pure Chemical Industries, Osaka, Japan) and the plasmid-transformed strain was grown on LB agar plates containing 25 μg/ml kanamycin (Sigma-Aldrich). *Vibrio harveyi* BB170 (kindly provided by Dr. B. L. Bassler, Princeton University) was cultured aerobically at 30°C on Marine Agar plates (Becton Dickinson).

### Construction of the *luxS*-deficient mutant

**Fig 1:**
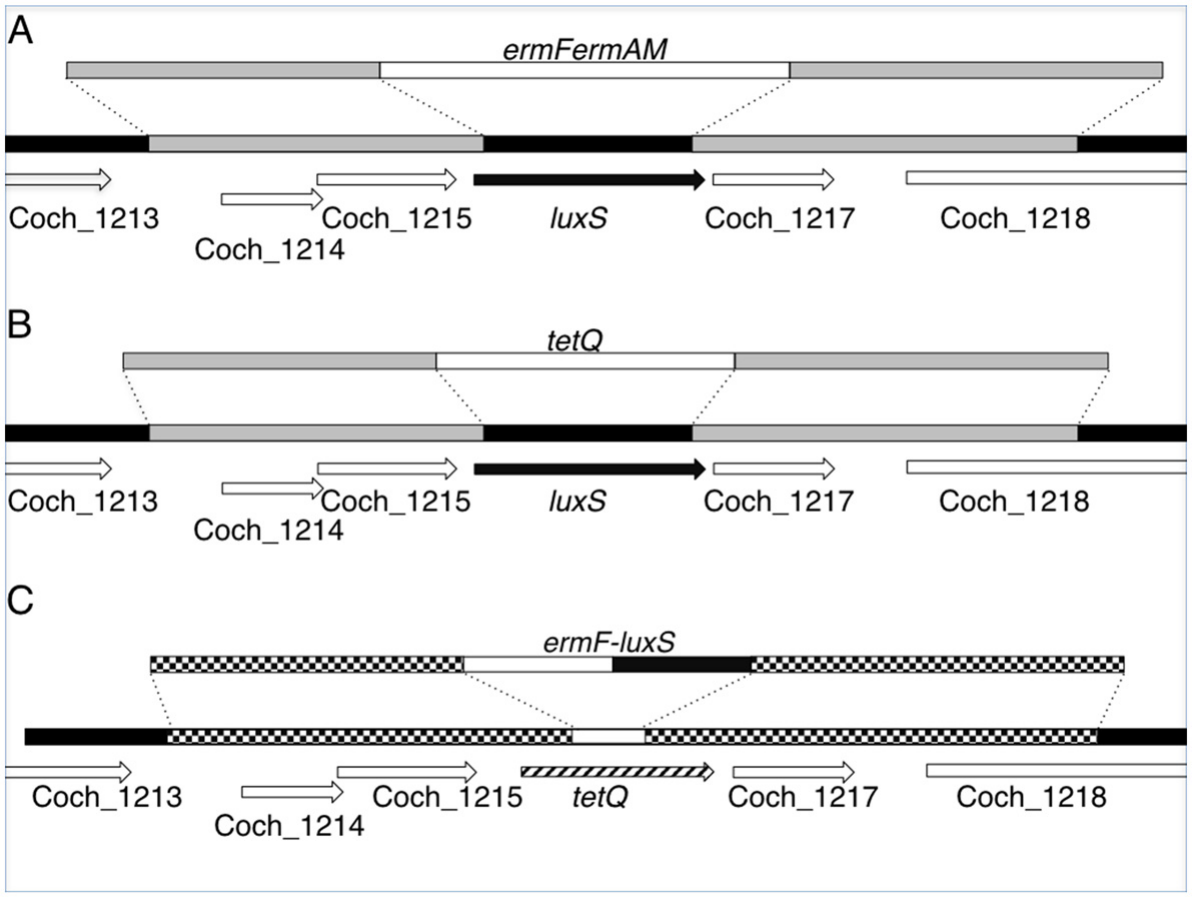
Schematic of inactivation and complementation of *luxS*. (A) Inactivation of *luxS by* using an *ermFermAM* cassette. The sequences that flanked the 5′- and 3′- ends of *luxS* (Coch_1216) were amplified with primers Capno1 and 2, and primers Capno3 and 4, respectively. The *ermFermAM* cassette was inserted between the amplified fragments and cloned. The plasmid was linearized and introduced into *C*. *ochracea* ATCC 27872 by electroporation. The resultant *luxS*∷*ermFermAM* strain was named TmAI2. (B) Inactivation of *luxS by* using *tetQ*. The *tetQ* fragment was inserted between the sequences that flanked the 5′- and 3′- ends of *luxS* by using the PCR-based overlap extension method and self-ligattion of the fragment, and then amplified in *E*. *coli*. The resultant plasmid was linearized and introduced into *C*. *ochracea* ATCC 27872 by electroporation. The resultant *luxS*∷*tetQ* strain was named LKT7. (C) Complementation of *luxS*. An *ermF-luxS* fragment was constructed by using the PCR-based overlap extension method to express both genes under control of the *ermF* promoter, and the fragment was cloned into the center of the *tetQ* gene in pAITQ. The resultant plasmid was linearized and introduced into *C*. *ochracea* LKT7 by electroporation. The resultant *tet*∷*ermF-luxS* strain was named luxS-C3.

To investigate the effect of LuxS on biofilm formation by *C*. *ochracea* ATCC 27872, we disrupted the *luxS* gene. We obtained the sequence of Coch_1216, which was annotated as *luxS* in the genome sequence *of C*. *ochracea* ATCC 27872 (NC_013162.1) was obtained from the NCBI database (http://www.ncbi.nlm.nih.gov/). The sequences that flanked the 5′- and 3′- ends of Coch_1216 were amplified from *C*. *ochracea* ATCC 27872 genomic DNA with primers Capno1 and 2, and primers Capno3 and 4 (Table 2), respectively, by using a Thermal cycler C1000 (Bio-Rad Laboratories, Hercules, CA, USA). The *ermFermAM* cassette was amplified from pVA2198 with primers EMU2 and EMD2 (Table 2), and inserted into the fragments amplified from *C*. *ochracea* by using a PCR-based overlap extension method described by Horton et al. [21]. The DNA sequences of the obtained fragments (Fig 1A) were confirmed, by using an ABI PRISM 3100 Genetic Analyzer (Applied Biosystems, Foster City, CA, USA), and then cloned into pCR2.1. The resultant plasmid, pAI2, was linearized by digestion with *Eco*RI and introduced into *C*. *ochracea* ATCC 27872 by electroporation: a 100-ml culture of mid-logarithmic–phase *C*. *ochracea* cells (optical density at 550 nm, [OD_550_], 0.3–0.6) was harvested, washed three times with ice-cold distilled water, and suspended in 0.2 ml 10% glycerol. Then, 2 μl of plasmid DNA (2 μg/μl) was mixed with 20 μl of the cell suspension. The mixture was incubated at room temperature for 1 min, and then transferred to a 0.1-cm electroporation cuvette (Bio-Rad). One pulse was delivered from a Gene Pulser II (Bio-Rad) at settings of 1.8 kV, 25 μF, and 250 Ω. Immediately after electroporation, the cells were suspended in 1 ml of 1.0 x TS broth and incubated overnight at 37°C under anaerobic condition. The cells were then plated onto BAPs containing 10 μg/ml erythromycin and incubated for 7–10 days at 37°C under anaerobic conditions to obtain a colony with a *C*. *ochracea*-like shape. The disruption of the Coch_1216 sequence in this TmAI2 mutant was confirmed by Southern blot analysis and DNA sequencing (data not shown).

**Table 2:** Primers used in this study.

Primer	Sequence (5′–3′)
Capno1	5′-GGGTAGTTTATTAGGCATCATACG-3′
Capno2	5′-TGTTGCAAATACCGATGAGCCCATTTTGTTTAGGTAAGAGATGATACC-3′
Capno3	5′-CGTTACTAAAGGGAATGTAGTATCCCAAGTAAAATATGAATACAATG-3′
Capno4	5′-AAGCGATGTGAGAGGTTGCCAAGGC-3′
Capno4b	5′-AAGCGATGTGAGAGGTTGCCAAGGCGTTAAGTGC-3′
EMD2	5′-GCTCATCGGTATTTGCAACATCATAG-3′
EMU2	5′-CTACATTCCCTTTAGTAACGTGTAACTTTC-3′
TetQWP	5′-AATCCTGCTGACCTTGTTTATGTCTTG-3′
TetQUP	5′-TTATTTTGATGACATTGATTTTTGGAACAT-3′
Capno3T	5′- CATCAAAATAAAATATGAATACAATGATTTTACAAGAACCCACT-3′
ermFF	5′-GCTCATCGGTATTTGCAACATCATAG-3′
LuxCErmFU	5′-CTACGAAGGATGAAATTTTTCAGGGACAAC-3′
LuxCD2	5′- AAAATTTCATCCTTCGTAGCTCTTACCTAAACAAAATGGAAAGAATAG-3′
LuxCU3	5′-TTACTTGGGATAATTCAGATTTTTATCGGTGAGGTGAT-3′
ermFluxF	5′-CAAAATGTTGTCGATGCTCATCGGTATTTGCAAC-3′
ermFluxR	5′-TACAATCGCGATATCTTACTTGGGATAATTCAGATTT-3′
LuxCKOF	5′-GATATCGCGATTGTAGAGGATATGGATGA-3′
LuxCKOR	5′-ATCGACAACATTTTGCATAAACAGGACATCT-3′

To investigate the effect of *luxS* on biofilm formation directly, we constructed another strain with a mutant *luxS gene* and performed complementation experiments. The sequences that flanked the 5′- and 3′- ends of Coch_1216 were amplified from pAI2 with primers Capno2 and Capno3T (Table 2) and the *tetQ* cassette was amplified from pKD 375 with primers TetQWP and TetQUP (Table 2) as described above. The *tetQ* fragment was connected with the Capno2-Capno3T fragment by the PCR-based overlap extension method and the resultant fragment was self-ligated and amplified in *E*. *coli* DH5α. The resultant plasmid, pAITQ, was linearized with *Not*I and the *luxS* gene was disrupted with this fragment (Fig 1B) as described above using BAPs containing 1.5 μg/ml tetracycline. The resultant mutant strain was designated *C*. *ochracea* LKT7. Inactivation of the *luxS* gene was confirmed by PCR and DNA sequencing (data not shown). To construct a *luxS*-complemented strain, we amplified the *ermF* fragment containing its promoter region from pVA2198 with primers ermFF and LuxCErmFU, and the *luxS* gene, including the ribosome binding site, was amplified with primers LuxCD2 and LuxCU3 from the genomic DNA of *C*. *ochracea* ATCC 27387. The fragments were connected by the PCR-based overlap extension method as described above. The *ermF-luxS* fragment was amplified with primers ermFluxF and ermFluxR and inserted into the center of the *tetQ* gene of pAITQ by using the primers LuxCKOF and LuxCKOR in an In-Fusion HD Cloning Kit (Takara-Bio, Kusatsu, Japan). The resultant plasmid, pLC2, was linearized and introduced into *C*. *ochracea* LKT7 (Fig 1C); the resultant complemented strain was designated *C*. *ochracea* luxS-C3.

### AI-2 assay

Production of AI-2 in the culture supernatants from *C*. *ochracea* strains was evaluated by using the reporter strain *V*. *harveyi* BB170. Culture supernatants of *C*. *ochracea* strains at OD_660_ = 0.3, 0.6, and 0.9 were collected by centrifugation at 10,000 *g* for 20 min and filtered through a 0.2-μm-pore-size filter. *V*. *harveyi* BB170 was incubated in autoinducer bioassay medium [22] containing the culture supernatant of *C*. *ochracea* strains for 2.5 h, and bioluminescence was measured with an Auto-LUMI-Counter 1422EX (Microtech-Nichion, Funabashi, Japan). AI-2 levels were calculated as the ratio of the *V*. *harveyi* bioluminescence intensity after the addition of culture supernatant to the intensity obtained after the addition of TS broth alone.

### Quantification of the mass of each biofilm

Overnight cultures of *C*. *ochracea* strains were diluted with 1.0 x TS broth or 0.5 × TS broth to an OD_660_ of 0.02, and 2-ml aliquots of the cell suspensions were inoculated into each well of 12-well polystyrene cell culture plates (BD Falcon, Franklin Lakes, NJ, USA), and incubated for 24 h or 48 h. The mass of each biofilm was quantified by staining with crystal violet as described previously [23]. In brief, the culture medium containing planktonic cells was removed, and the wells were washed with distilled water. Adherent bacteria were stained with 0.1% crystal violet and rinsed twice with distilled water. The dye bound to each biofilm was then extracted with 95% ethanol and quantified as the OD_595_ on a microplate reader (Spectra MAX M5; Molecular Device, Sunnyvale, CA, USA).

### Analysis of biofilm structure by use of confocal scanning laser microscopy and scanning electron microscopy

Biofilms of *C*. *ochracea* ATCC 27872 and TmAI2 formed on glass surfaces were analyzed by using confocal scanning laser microscopy (CSLM) as follows. *C*. *ochracea* ATCC 27872 or TmAI2 was inoculated into the wells of a 12-well polystyrene plates, in which each well contained a coverglass and 0.5 × TS broth, and incubated for 48 h. The coverglasses were washed once in distilled water, and the biofilms that formed on the coverglasses were fixed with 4% paraformaldehyde solution, gently washed twice with phosphate-buffered saline (pH 7.4, PBS), and then stained with propidium iodide (10 μM) for 2 h. The biofilms were examined under an LSM5 DUO microscope (Carl Zeiss MicroImaging, Göttingen, Germany) with a 63x oil immersion objective. A series of 20–25 Z-stack images were scanned in increments by using excitation wavelengths of 405–450 nm and 532 nm. Images were analyzed with ZEN 2008 software (Carl Zeiss MicroImaging). Where appropriate, Z stacks of the X–Y sections of the CSLM were processed to render a three-dimensional image by using the IsoSurface function of Imaris 7.0.0 software (Bitplane AG, Zürich, Switzerland).

The height of the biofilm was measured in six randomly selected images by using Zen 2009 software (Carl Zeiss MicroImaging) and the means were calculated. To evaluate the roughness of the biofilm structure, we calculate the percentage volume occupied by *C*. *ochracea* in the total biofilm volume from six randomly selected integrated CSLM images by using Imaris 7.4.0 software (Bitplane AG; Zürich, Switzerland).

Biofilms of *C*. *ochracea* ATCC 27872 and TmAI2 formed on polystyrene surfaces were also analyzed by using scanning electron microscopy (SEM) as follows. *C*. *ochracea* ATCC 27872 or TmAI2 was inoculated into 12-well polystyrene plates in which each well contained a 5-mm x 5-mm coverglasses (Matsunami Glass, Osaka, Japan) and 1.0 or 0.5 × TS broth, and incubted for 48 h. The biofilms that formed on the coverglasses were washed once in distilled water, and the biofilms that formed on them were fixed with 4% paraformaldehyde solution at 20°C overnight. After dehydration through a graded ethanol series, the polystyrene tips were air dried and sputter coated with gold by using an ESC-101 SEM sample coating system (ELIONIX, Tokyo, Japan). The biofilms were then examined at ×500 to ×3000 magnification under an electron microscope (SU6600, Hitachi, Tokyo, Japan).

### Adherence activity of *C*. *ochracea*

Measurement of adherence by *C*. *ochracea* cells to polystyrene plates was performed by an enzyme-linked immunosorbent assay (ELISA)–based assay as described previously [24] with minor modifications. Briefly, overnight cultures of *C*. *ochracea* cells were incubated in a 96-well polystyrene plate for 1 h and washed twice with PBS. After blocking, rabbit anti-serum against *C*. *ochracea* whole cells was added, and the plates were incubated for 1 h. After washing with PBS, goat anti-rabbit IgG conjugated with horseradish peroxidase (Bio-rad) was added and allowed to react for 1 h, and the plates were then washed twice with PBS. Color development was carried out with ABTS Peroxidase Substrate (KPL, Gaithersburg, MD, USA) and absorbance was measured at 405 nm with a microplate reader (Spectra MAX M5).

### Effect of extrinsic AI-2 on biofilm formation

To investigate whether extrinsic AI-2 is involved in biofilm formation, *C*. *ochracea* was grown in a two-compartment system as described previously [25]. In addition, we prepared a mixed-culture of *C*. *ochracea* ATCC 27872 and TmAI2, and evaluated the effect of the culture supernatant of the wild-type strain (ATCC 27872). In the two-compartment system, each well of a 12-well polystyrene cell culture plate was separated into two compartments by using a 0.4-μm pore-size Anopore membrane (BD Falcon cell culture inserts, Becton, Dickinson). *C*. *ochracea* wild-type and TmAI2 were separately inoculated into the lower or upper compartments in all 4 combinations and incubation at 37°C for 48 h under anaerobic conditions. After, the membrane units were removed, the amounts of biofilm in the lower compartments were evaluated as described above.

In the mixed-culture experiments, overnight cultures of *C*. *ochracea* ATCC 27872 or TmAI2 were diluted as described under “Quantification of the biomass of each biofilm”. Each well of a 96-well polystyrene cell culture plate was inoculated with 200 μl of the wild-type strain alone, 200 μl of TmAI2 alone, or 100μl each of the wild-type strain and TmAI2. After incubation at 37°C for 48 h under anaerobic conditions, the amounts of biofilm were evaluated as described above.

To investigate the effect of the culture supernatant of *C*. *ochracea* ATCC 27872 or TmAI2 on biofilm formation, culture supernatant was collected from overnight cultures (OD_660_≈0.7) of *C*. *ochracea* ATCC 27872 or TmAI2 and filtered through an 0.22-μm filter (Merck Millipore, Billerica, MA, USA). *C*. *ochracea* ATCC 27872 and TmAI2 were diluted as described under “Quantification of the biomass of each biofilm”, 100-μl aliquots were inoculated into each wells of a 96-well polystyrene cell culture plate, and then 100 μl of the culture supernatants was then added. After incubation at 37°C for 48 h under anaerobic conditions, the amounts of biofilm were evaluated as described above.

### Statistical analysis

The Student’s t-test was used to compare the quantity of biofilm formation and adherence activity between strains and conditions. The effect of extrinsic AI-2 on biofilm formation was compared with a one-way ANOVA followed by a Dunnett’s multiple comparison test. *P* values less than 0.05 were considered statistically significant. All tests were conducted in Prism 5.0f software (GraphPad Software, Inc., La Jolla, CA, USA).

## Results

### AI-2 production by *C*. *ochracea* TmAI2

**Fig 2:**
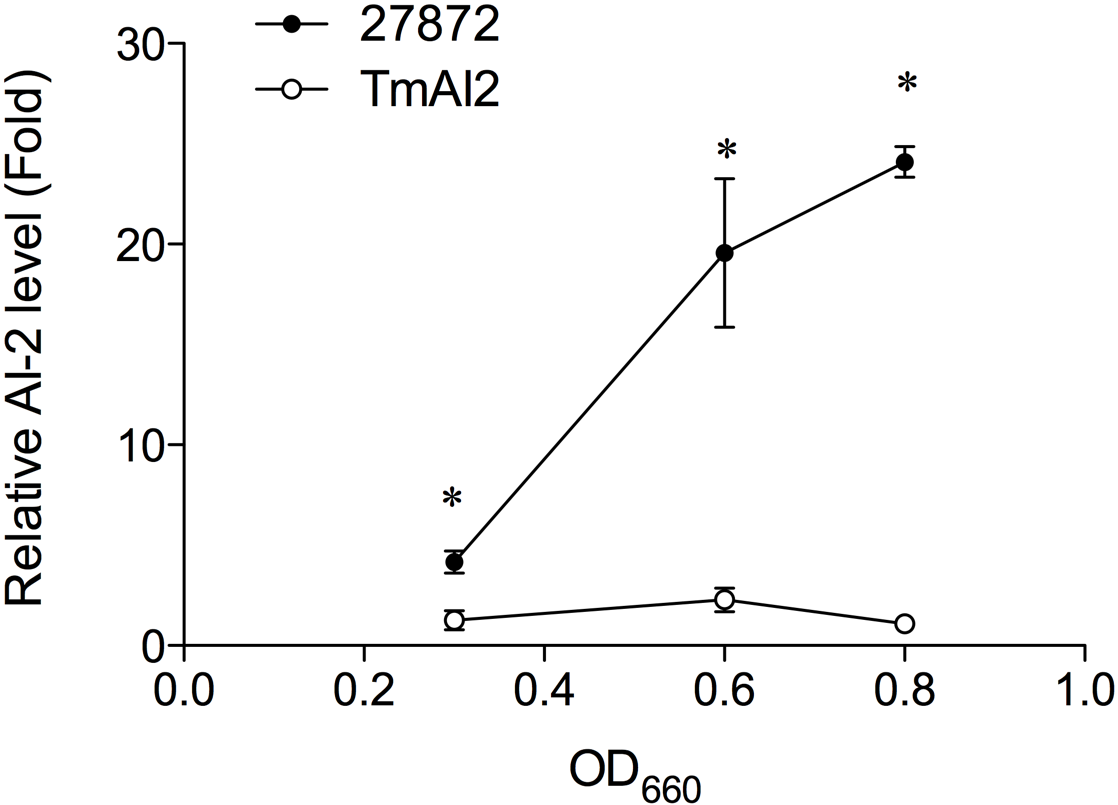
AI-2 production from *C*. *ochracea*. AI-2 levels are expressed as the ratio of the *V*. *herveyi* bioluminescence intensity after addition of culture supernatant to the intensity obtained after addition of TS broth alone. 27872; *C*. *ochracea* ATCC 27872 (wild type), TmAI2; *C*. *ochracea* TmAI2. *, *P* < 0.05 compared with TmAI2.

To verify that Coch_1216 codes for LuxS in *C*. *ochracea*, the levels of AI-2 produced by *C*. *ochracea* ATCC 27872 (wild type) and TmAI2, which has a disrupted Coch_1216 sequence, were measured. The AI-2 level (as measured by *V*. *harveyi* BB170 bioluminescence intensity) after addition of wild-type culture supernatant was 4 to 24 times that after incubation with TS broth alone, while that after addition of TmAI2 culture supernatant was the same as that after incubation with TS broth alone (Fig 2). The difference in AI-2 levels between TmAI2 and wild type was statistically significant at each growth phase examined. Therefore, Coch_1216 codes for LuxS in *C*. *ochracea*.

### Gliding motility and biofilm formation of *C*. *ochracea* TmAI2

**Fig 3:**
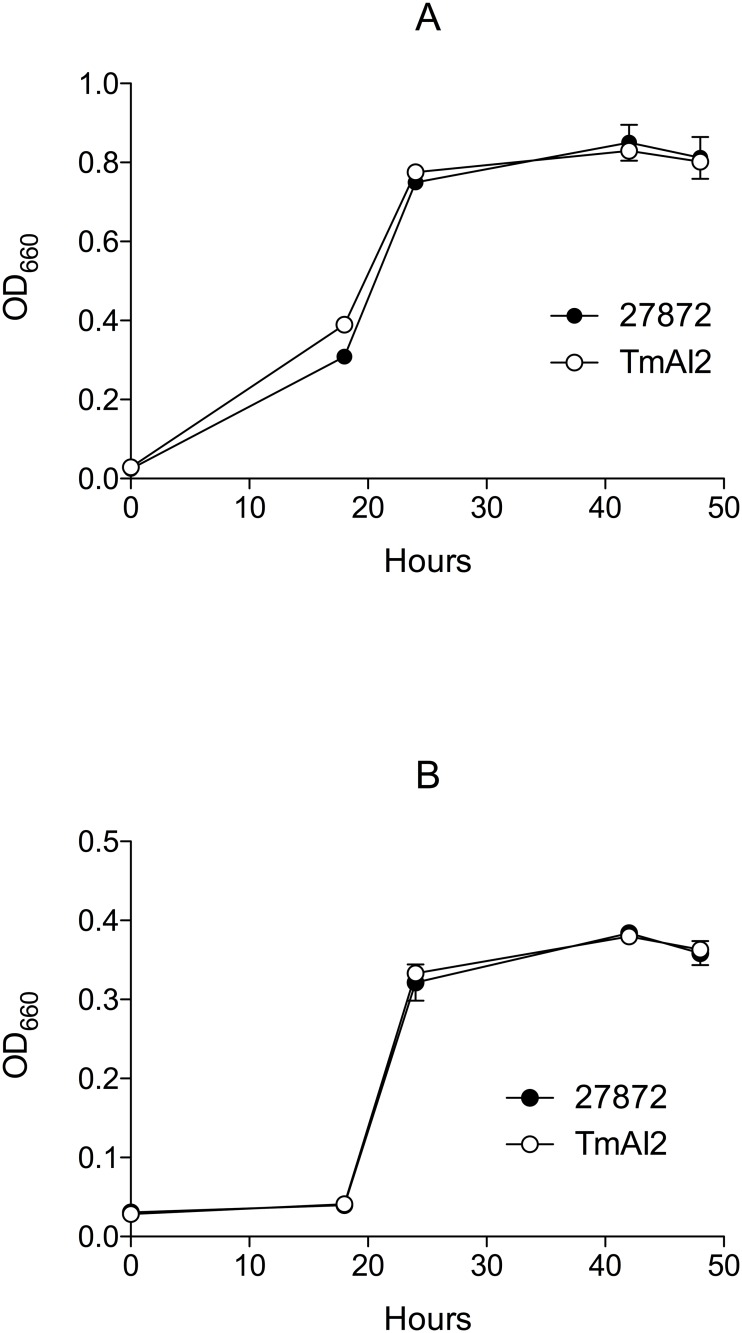
Growth curves of the *C*. *ochracea* wild-type and TmAI2 strains. *C*. *ochracea* ATCC27872 and TmAI2 were cultured in TS broth at 37°C, under anaerobic conditions, and their growth in 1.0 x TS broth (A) and 0.5 × TS broth (B) was measured as OD_660_. Data are means ± SD (n = 3).

The growth of *C*. *ochracea* TmAI2 was similar to that of the wild-type strain during the period from 0 to 48 h in 1.0 x TS broth (Fig 3A) or 0.5 × TS broth (Fig 3B). *C*. *ochracea* has gliding motility, which permits spreading colony formation. The colony morphology and spreading size of *C*. *ochracea* TmAI2 on BAPs resembled that of the wild-type strain (data not shown), indicating that the gliding motility of *C*. *ochracea* was not affected by inactivation of the *luxS* gene.

**Fig 4:**
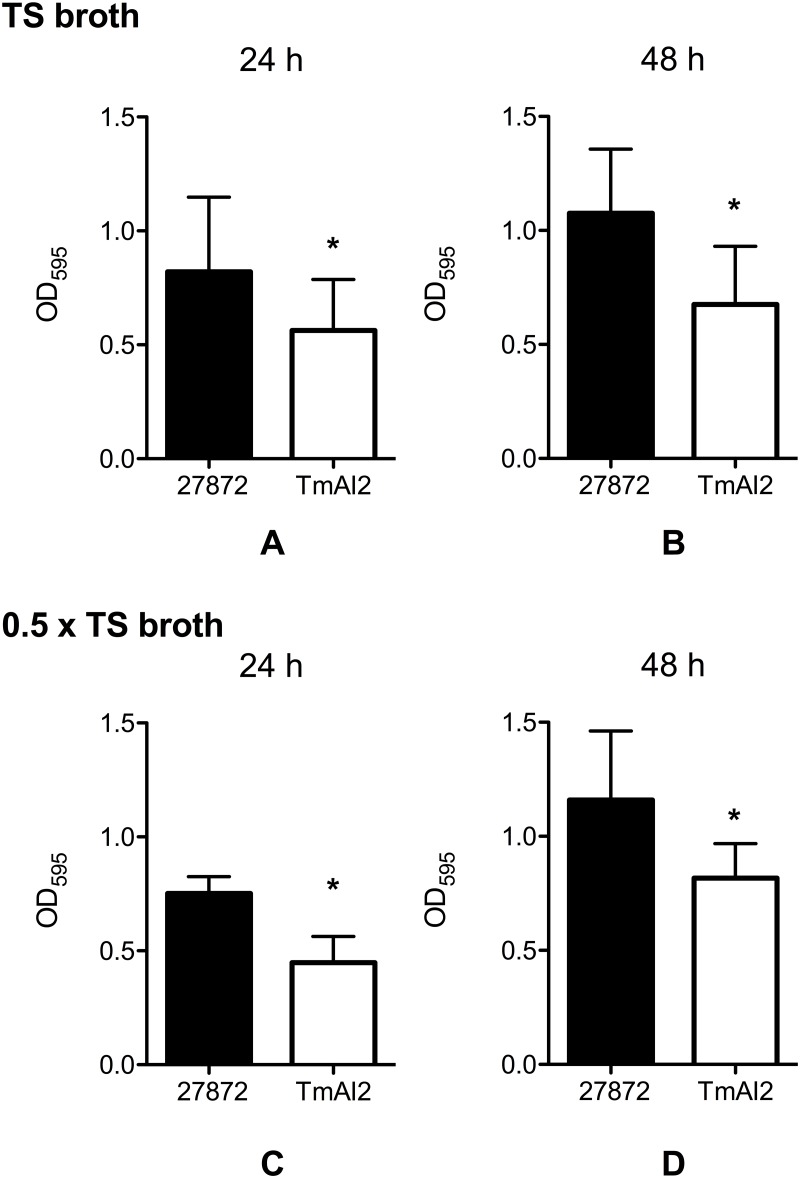
Biofilm formation by *C*. *ochracea* wild type and TmAI2. *C*. *ochracea* ATCC27872 and TmAI2 were incubated in (A) 1.0 x TS broth for 24 h, (B) 1.0 x TS broth for 48 h, (C) 0.5 × TS broth for 24 h, or (D) 0.5 × TS broth for 48 h, in a 12-well plate under anaerobic conditions. Biofilm formation was then assayed by crystal violet staining. Data are presented as means ± SD (n = 10). *, *P* < 0.05 compared with wild type.

We measured the mass of the biofilms produced by *C*. *ochracea* wild-type and TmAI2 at 24 h and 48 h, in both 1.0 x TS broth and 0.5 × TS broth (Fig 4). The mass of the TmAI2 biofilm was significantly less than that of the wild-type biofilm at both 24 h (Fig 4A and 4C) and 48 h (Fig 4B and 4D). The mass of the biofilm formed in 0.5 × TS broth (Fig 4C and 4D) was similar to that of the biofilm formed in the 1.0 x TS broth (Fig 4A and 4B).

### CSLM and SEM analysis of biofilm architecture

**Fig 5:**
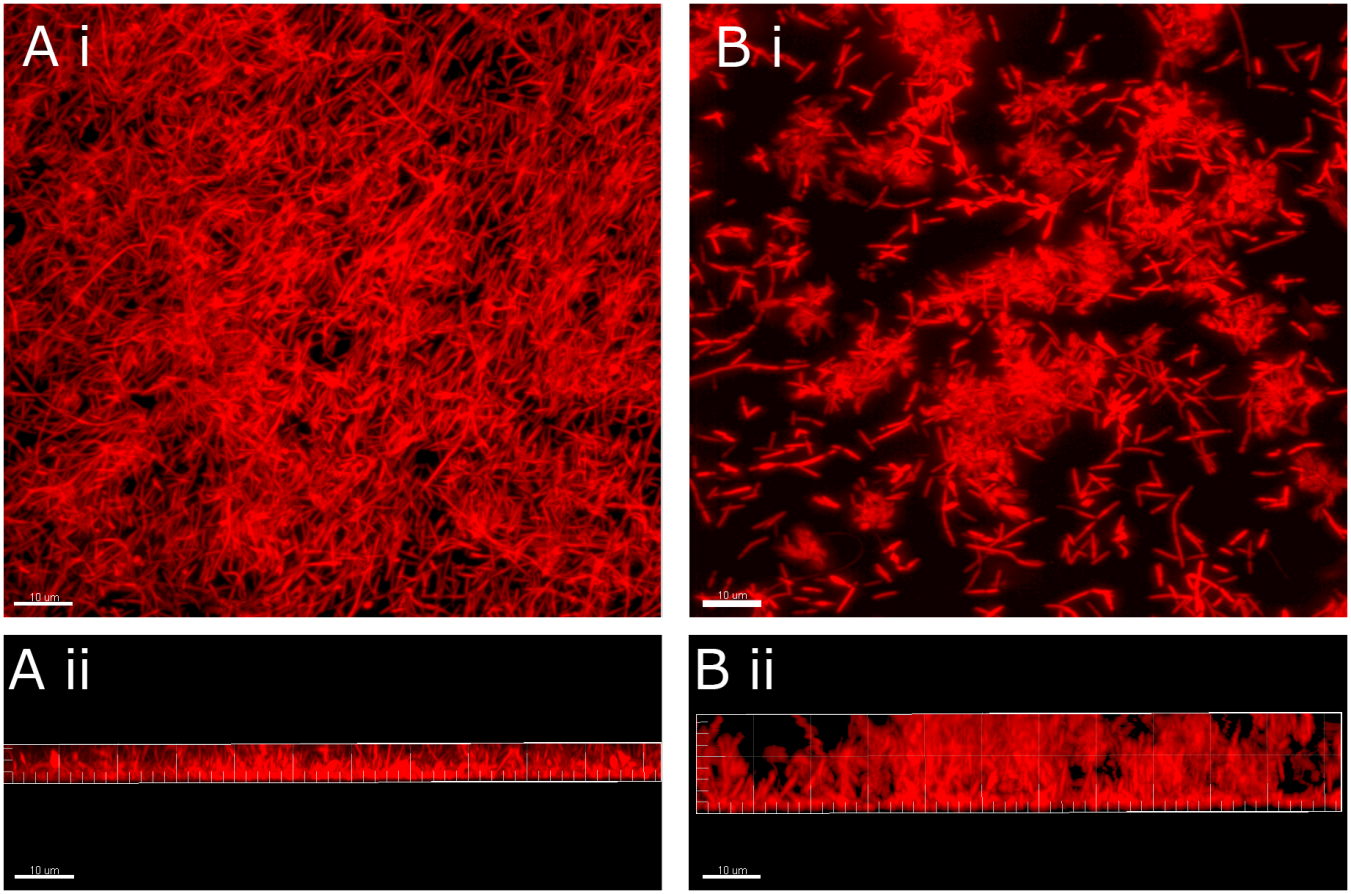
Representative CSLM images of biofilms of *C*.*ochracea* wild type and TmAI2. *C*. *ochracea* ATCC27872 (A) and TmAI2 (B) were incubated in 12-well polystyrene plates containing coverglass plates for 48 h, and CSLM images of the cells that attached to the coverglass were obtained. The upper panels (i) show each **x**-**y** images, and the lower panels (ii) show the center of each **x**-**z** reconstructions. Scale bars, 50 μm.

**Fig 6:**
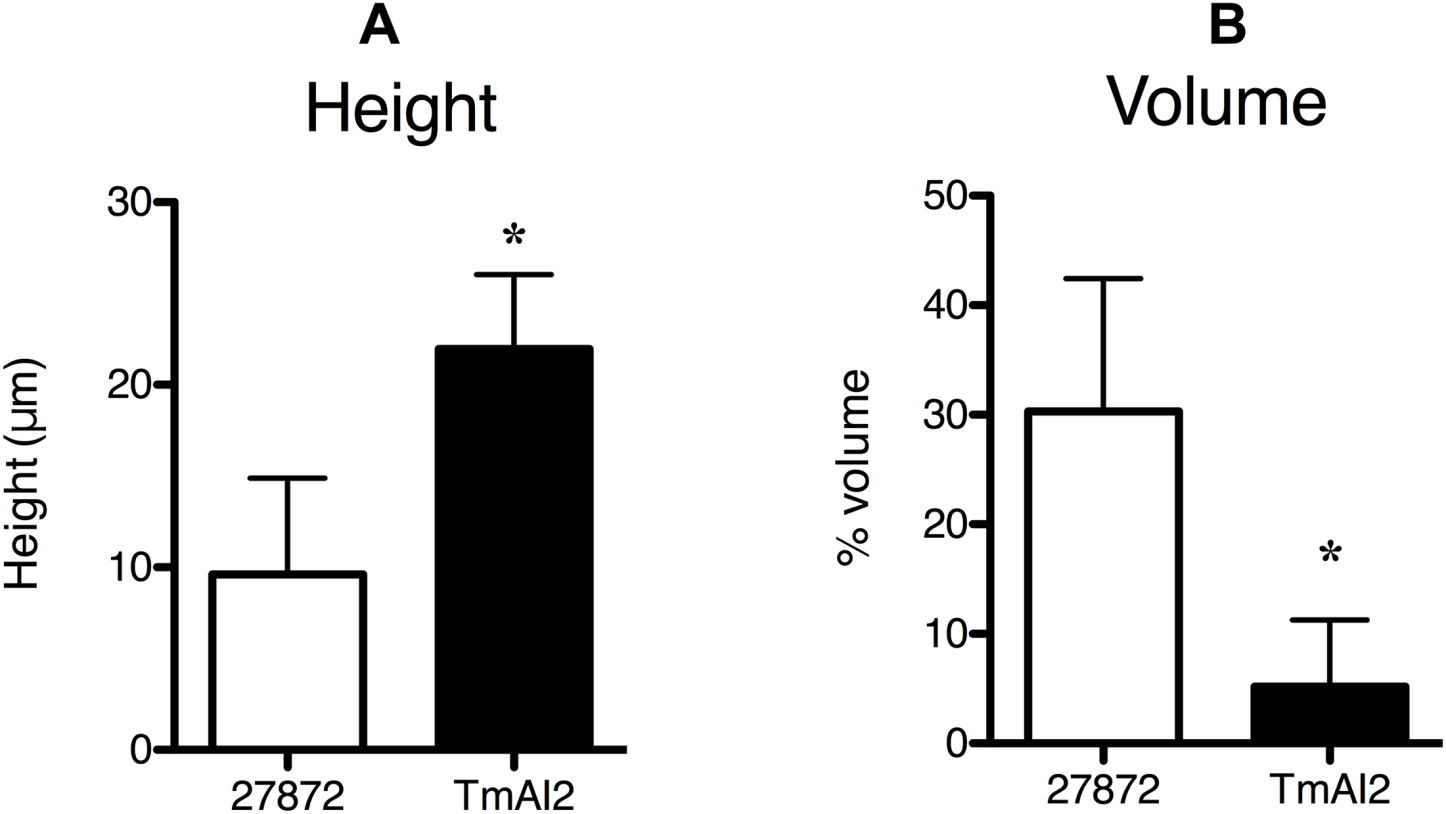
Quantitative analysis of the biofilm structure of *C*. *ochracea*. The height (A) and % volume occupied by *C*. *ochracea* in total volume (B) are shown. The height was evaluated by using Zen 2009 software and the volume by use of IMARIS software. Data are presented as means ± SD of OD_405_. The valuation was performed at six points. *, *P* < 0.05 compared to wild type.

According to the results of CSLM analysis, the height of biofilm formed by *C*. *ochracea* TmAI2 (Fig 5B) was greater than that formed by the wild-type strain (Fig 5A). Quantitative analysis showed that this difference in height was statistically significant (Fig 6A). In addition, the biofilm formed by *C*. *ochracea* TmAI2 (Fig 5B) appeared to be rougher in architecture than that formed by the wild-type strain (Fig 5A). Further analysis showed that the volume occupied by TmAI2, as a percentage of total biofilm volume, was significantly smaller than that occupied by the wild-type strain (Fig 6B), confirming the rough structure of the biofilm organized by *C*. *ochracea* TmAI2.

**Fig 7:**
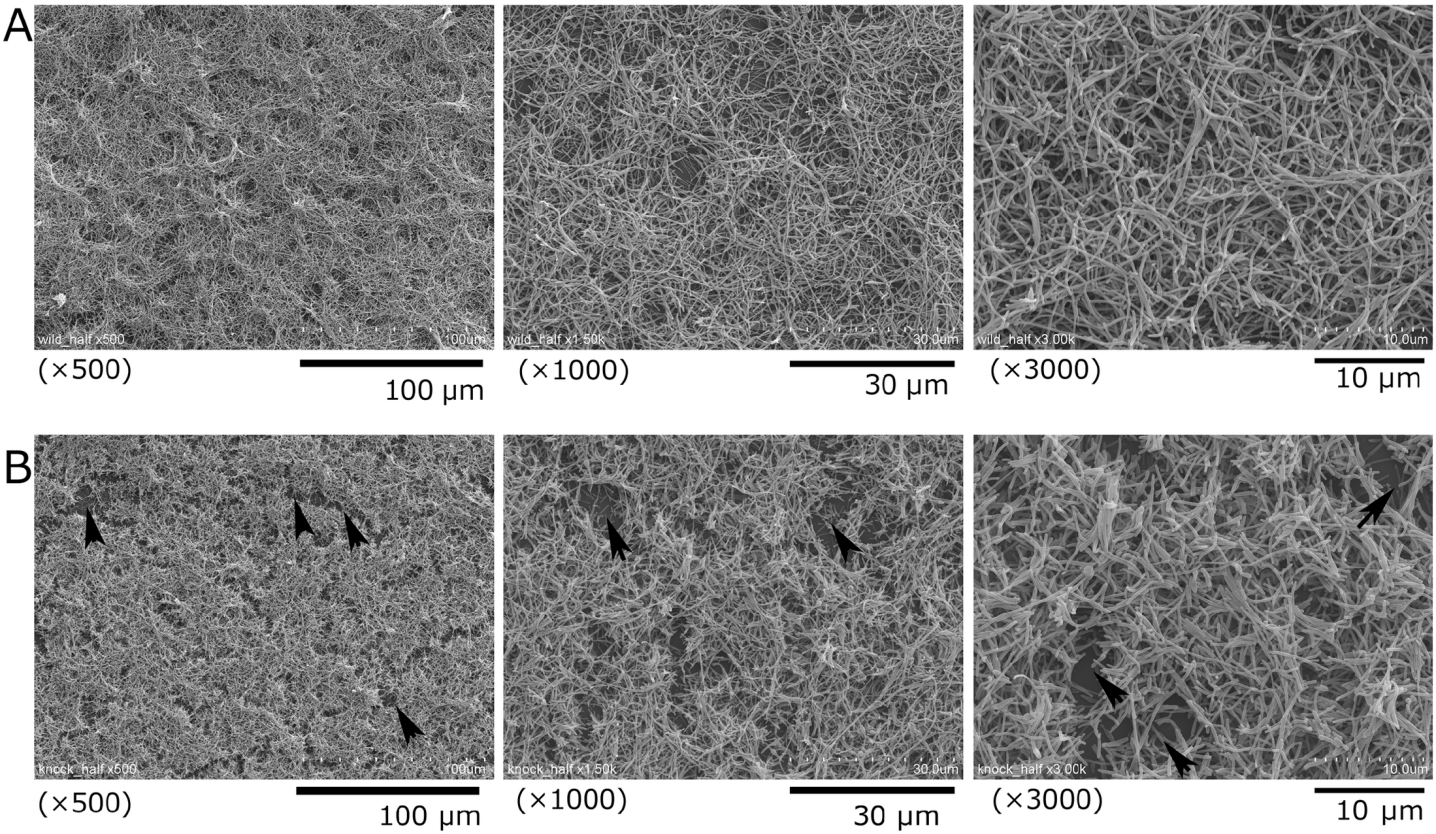
Representative SEM images of biofilms of *C*.*ochracea* wild type and TmAI2. *C*. *ochracea* ATCC27872 (A) and TmAI2 (B) were incubated anaerobically on coverglasses in 12-well polystyrene plates for 48 h. Representative images of the biofilms are shown at the indicated magnifications. Arrowheads indicate partially vacant spaces.

In SEM analysis, at low magnification, the biofilms formed by *C*. *ochracea* wild-type (Fig 7A) and TmAI2 (Fig 7B) appeared similar to each other; however, at high magnification, partially vacant spaces were apparent in the biofilm formed by TmAI2 only. This finding is consistent with the rough structure observed by the CSLM analysis.

### Adherence by *C*. *ochracea TmAI2*

**Fig 8:**
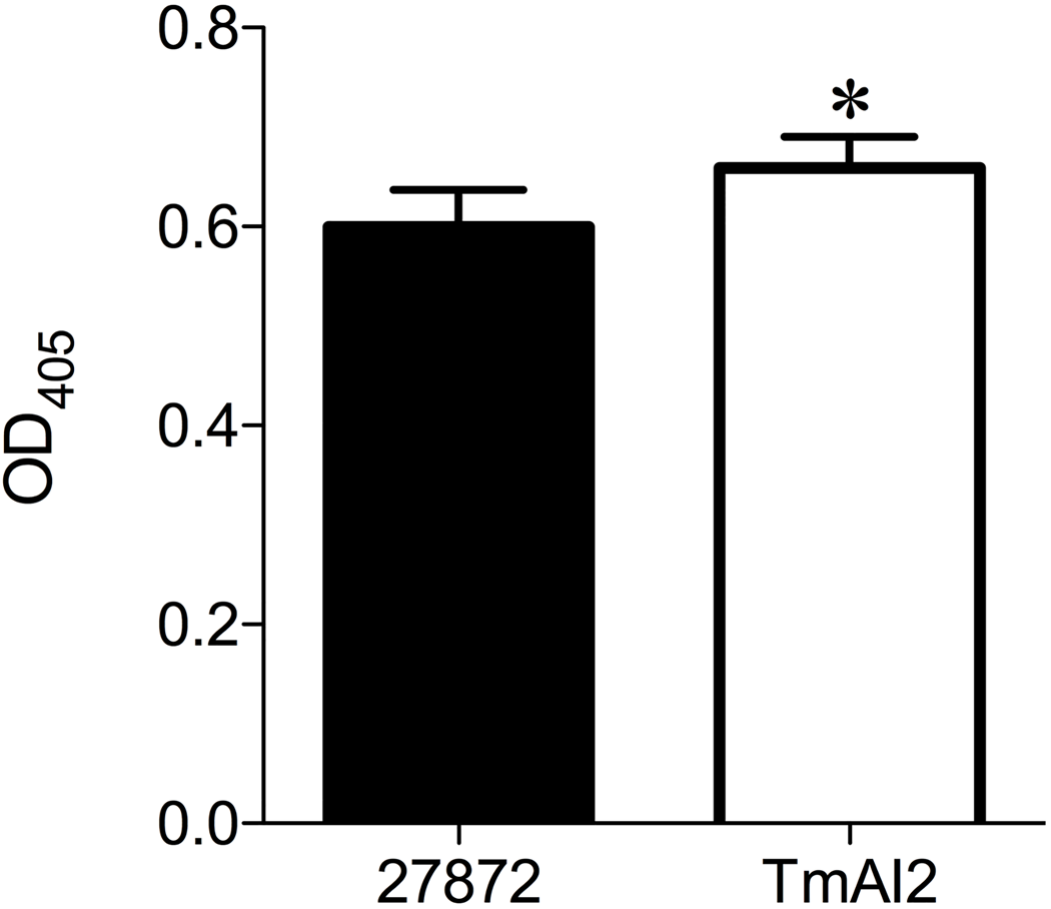
Adherence of *C*. *ochracea* to polystyrene plates. *C*. *ochracea* ATCC27872 and TmAI2 were incubated in 96-well polystyrene plates for 1 h. Adherence was measured at OD_405_ with an ELISA-based assay. Data are presented as means ± SD (n = 12).

To clarify the involvement of cell adherence to the hard surfaces in biofilm formation, we investigated the adherence of *C*. *ochracea* wild-type and TmAI2 to polystyrene plates. The adherence of TmAI2 was slightly but significantly stronger than that of the wild-type strain (Fig 8), indicating that the difference in biofilm formation was not due to the initial step of adhesion.

### Effect of complementation on biofilm formation

**Fig 9:**
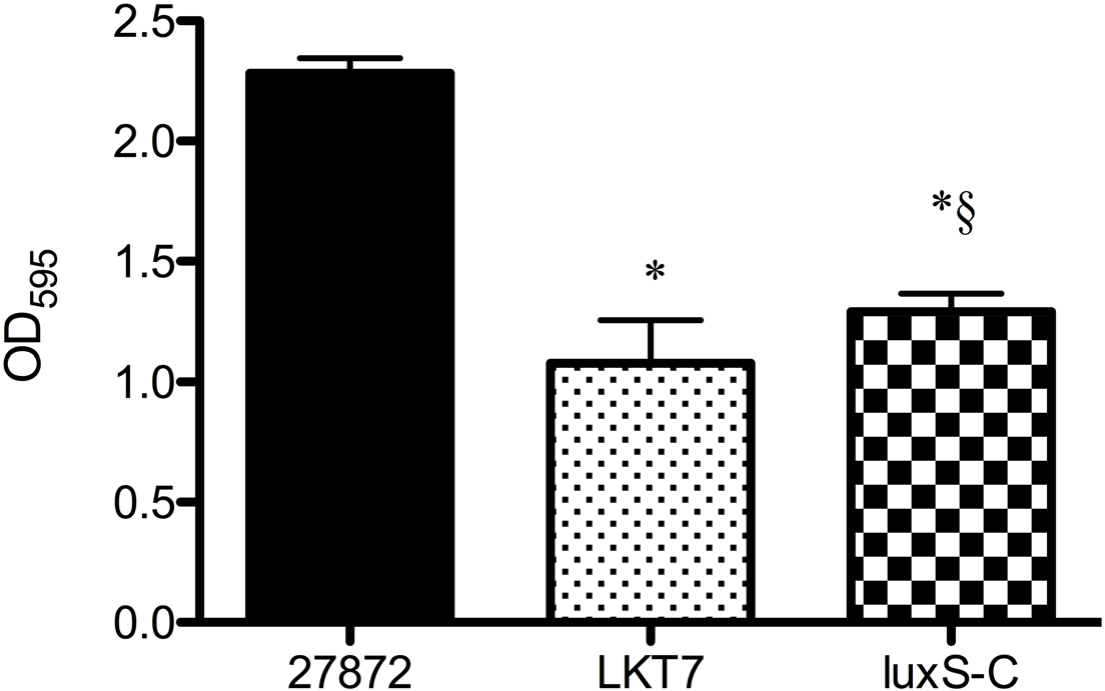
Effect of *luxS* complementation on biofilm formation by *C*. *ochracea*. *C*. *ochracea* ATCC27872, LKT7 (*luxS*∷*tetQ*), and luxS-C3 (*luxS* complemented strain) were incubated in TS broth for 48 h under anaerobic conditions. Biofilm formation was then assayed by means of crystal violet staining. Data are presented as means ± SD (n = 6) *, *P* < 0.05 compared with wild type, § *P* < 0.05 compared with the LKT7.

To clarify the involvement of *luxS* in biofilm formation, a *luxS* complemented strain was constructed. Firstly, a *luxS*-deficient mutant, *luxS*∷*tetQ* (LKT7) was constructed with a *tetQ* cassette. Complementation was performed by inserting the *ermF-luxS* fragment into the center of the *tetQ* gene in the LKT7 strain. The growth of *C*. *ochracea* LKT7 and luxS-C3 was similar to that of the wild-type strain (data not shown). In the complemented strain, luxS-C3, *luxS* was expressed as an operon with *ermF*. The AI-2 activity in the was not detected from the culture medium of the LKT7 strain. The AI-2 activity in the culture medium of the luxS-C3 strain was 21.3% ± 1.27 of that of the wild-type strain. The biofilm formation of the LKT7 strain was significantly decreased compared with wild type strain, and that of the luxS-C3 was partially restored (17.8% relative to the reduction from wild type to the strain LKT7, Fig 9).

### Effect of extrinsic AI-2 on biofilm formation

**Fig 10:**
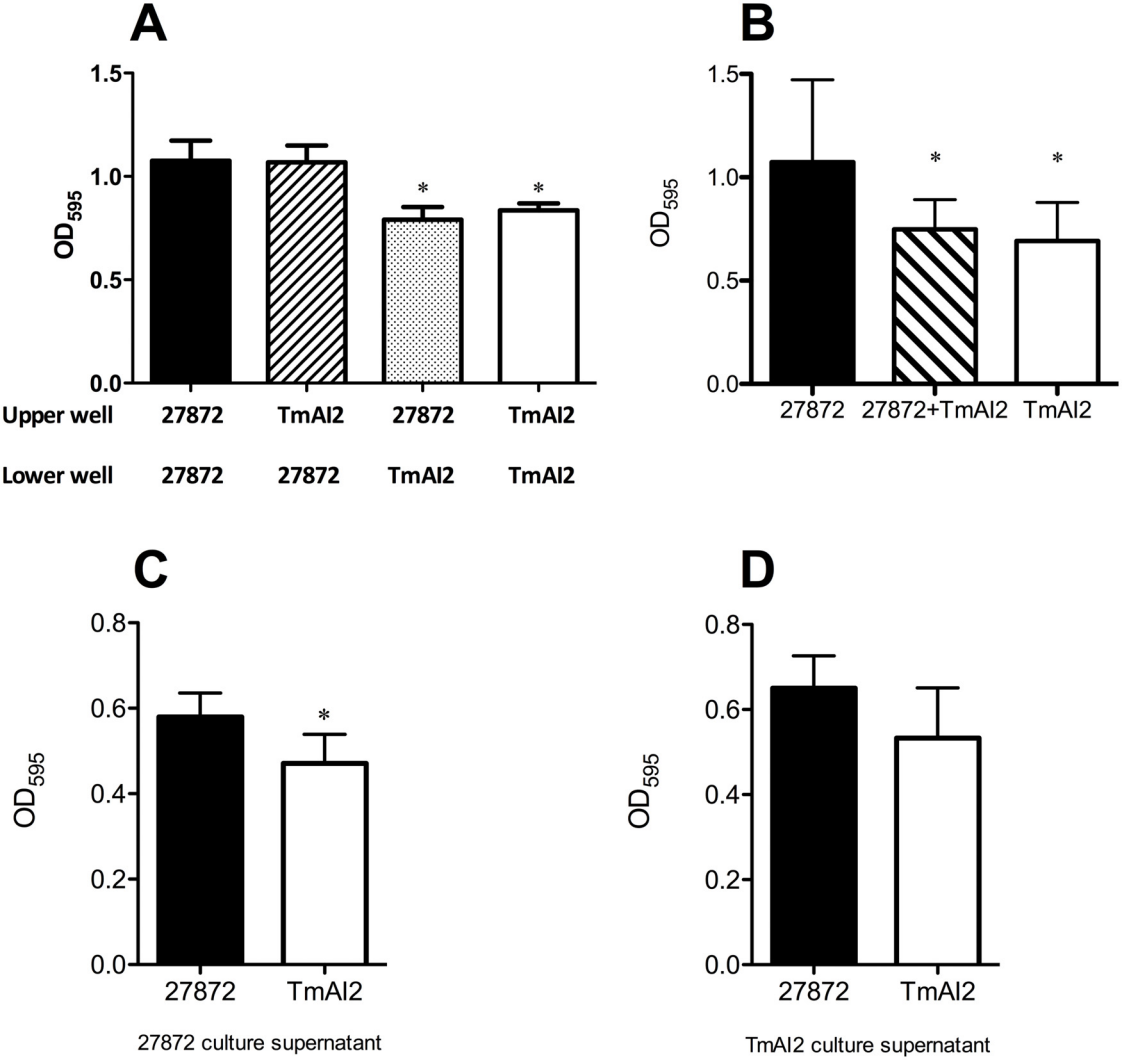
Effect of extrinsic AI-2 on biofilm formation by the *luxS*-deficient mutant and wild-type strain. (A) Assessment of biofilm formation by mutant and wild-type strains by using a two-compartment system. *C*. *ochracea* ATCC 27872 and TmAI2 were inoculated into TS broth in the indicated compartments (upper or lower well) in the wells of a 12-well polystyrene plate, and were then incubated for 48 h. Biofilm formation by the strain in the lower compartment of each well was stained with crystal violet staining. After rinsing and ethanol extraction, the mass of biofilm was quantified as OD_595_ of extracted stain. Data are presented as means ± SD (n = 10). *, *P* < 0.05 compared with the biofilm formation by *C*. *ochracea* ATCC 27872 in the lower compartment with *C*. *ochracea* ATCC 27872 in the upper compartment. (B) Effect of mixed-culture on biofilm formation by mutant and wild-type strains. *C*. *ochracea* ATCC 27872 and TmAI2 were inoculated into the wells of a 96-well plate, incubated for 48 h, and biofilm formation was stained with crystal violet staining. After rinsing and ethanol extraction, the mass of biofilm was quantified as OD_595_ of extracted stain. The data are presented as means ± SD (n = 21). * *P* < 0.05 compared to *C*. *ochracea* ATCC 27872 alone. (C) Effect of wild-type culture supernatant of the wild-type strain on biofilm formation by *C*. *ochrachea*. *C*. *ochracea* ATCC 27872 or TmAI2 cells were inoculated into the wells of a 96-well plate, and culture supernatant of *C*. *ochracea* ATCC 27872 was added. The plates were incubated for 48 h anaerobically, and then biofilm formation was stained with crystal violet staining. After rinsing and ethanol extraction, the mass of biofilm was quantified as OD_595_ of extracted stain. Data are presented as means ± SD (n = 6). * *P* < 0.05 compared with *C*. *ochracea* ATCC 27872 alone. (D) Effect of *luxS* mutant culture supernatant on biofilm formation by *C*. *ochrachea*. One hundred microliters of *C*. *ochracea* ATCC 27872 or TmAI2 were inoculated into the wells of a 96-well polystyrene plate, and culture supernatant of *C*. *ochracea* TmAI2 was added. Then the plates were incubated for 48 h anaerobically, and biofilm formation was stained with crystal violet staining. After rinsing and ethanol extraction, the mass of biofilm was quantified as OD_595_ of extracted stain. Data are presented as means ± SD (n = 6). * *P* < 0.05 compared with *C*. *ochracea* ATCC 27872 alone.

To investigate the effect of extrinsic AI-2 on biofilm formation, the wells of a 12-well polystyrene cell culture plate were partitioned into a two-compartment system. The levels of biofilm formation in the lower compartment are shown in Fig 10A. Biofilm formation by *C*. *ochracea* TmAI2 was not restored to the level of the wild-type strain, even after inoculation of the wild-type strain into the upper compartment. In 96-well polystyrene plates, mix-culture of *C*. *ochracea* wild-type and TamAI2 formed levels of biofilm intermediate between those of the wild-type and TamAI2 strains (Fig 10B). The addition of the culture supernatant of the wild-type strain (or of the negative control, TmAI2) did not restore the biofilm formation by TmAI2 (Fig 10C and 10D). These results indicate that extrinsic AI-2 had little effect on biofilm formation.

## Discussion

To clarify the role of LuxS in biofilm formation by *C*. *ochracea*, we created a *luxS*-deficient mutant of *C*. *ochracea* ATCC 27872, TmAI2. In the wild-type strain, the level of AI-2 production increased according to cell density. However, AI-2 production was not detected in the TmAI2 culture medium and the biofilm forming activity of TmAI2 was attenuated under all conditions tested. The production of one of the distant precursors of AI-2, a signaling molecule in the quorum sensing system, is catalyzed by LuxS, which has been identified in many Gram-positive and Gram-negative bacteria [26]. In dental plaque, AI-2 has been suggested to play an important role in interspecies communication [27]. Among oral Gram-negative rods, AI-2 production has been reported in *Porphyromonas gingivalis*, *Aggregatibacter actinomycetemcomitans*, *F*. *nucleatum*, *and Prevotella intermedia*, but not in *Capnocytophaga sputigena* [28]. Among these AI-2-producing strains, only *F*. *nucleatum* appears to lack a *luxS* homologue based on DNA sequencing [15]. We previously detected AI-2 production in *C*. *ochracea* [19], and the current results indicate that the product of Coch_1216 has LuxS activity.

There are two possible explanations for the observed attenuation of biofilm production by *C*. *ochracea* TmAI2 compared with the wild-type strain: a decrease in the initial adherence and a decrease in the biofilm formation after adherence. When we compared the adherence between the *C*. *ochracea* wild-type and TmAI2 strains, TmAI2 showed slightly stronger attachment to the polystyrene plate. Furthermore, the gliding motility was similar between the wild-type and mutant strains. These results suggest that the disruption of the *luxS* gene in TmAI2 reduced biofilm formation after adherence.

Biofilms of *C*. *ochracea* TmAI2 were rough in architecture with taller microcolonies compared with those of the wild-type strain. These characteristics resemble those previously reported in the *luxS*-inactivated strains of oral streptococci [29, 30]. For instance, biofilms of the *Streptococcus mutans luxS* deficient mutant are loose, hive-like, and have large gaps in the biofilm matrix [29]; and biofilms of the *Streptococcus gorndonii luxS* deficient mutant exhibit finger-like projections of cells that extend into the flow cell lumen [30]. In our results, *C*. *ochracea* adherence activity was slightly increased by disruption of *luxS*, suggesting that LuxS deficiency may affect microcolony formation after adherence in this bacterium. In the *Streptococcus mutans luxS* deficient mutant, glucosyltransferase B and C were upregulated and biofilm regulatory protein A was downregulated compared with that in the wild-type strain [25, 29]. Extracellular polysaccharide is important in biofilm formation by *S*. *mutans* [31]. *C*. *ochracea* reported to produce mannose rich extracellular polysaccharide [32], however the polysaccharide was isolated from culture supernatant and its involvement in biofilm formation has not reported. An extracellular polysaccharide-like structure was observed in biofilms of *S*. *mutans* [25, 29], while that was not observed by the SEM analysis here (Fig 7). Therefore, although the rough structure of the biofilms formed by the *luxS* deficient strains of *C*. *ochracea* and oral streptococci are similar, the main factors contributing to biofilm formation by these bacteria may be different. Proteinaceous materials and extracellular DNA are also involved in biofilm formation as a matrix [33]. Orthologues of genes encoding the Type 9 secretion system, which involves the export of surface proteins of *Flavobacterium johnsoniae* and *P*. *gingivalis* [34], were detected in the genome sequence of *C*. *ochracea* [35]. It is possible that protein exported by the Type 9 secretion system is involved in *C*. *ochracea* biofilm formation. The present findings suggests that inactivation of *luxS* affects factors involved in microcolony formation in *C*. *ochracea*, although further analysis of the gene regulation involved in microcolony formation in this microorganism is required.

In the present study, the biofilm formation of *C*. *ochracea* TmAI2 and LKT7 was significantly reduced. Biofilm formation ability was partially restored in the *luxS*-complemented strain, suggesting that *luxS* is involved in biofilm formation. The AI-2 production of the luxS-C3 strain was restored approximately 20% of that in the wild type, and biofilm formation of the Lux-C3 strain was also restored by approximately 17% of the reduction of the biofilm formation from the wild type strain to the LKT7 strain. The low degree of restoration could be attributed to the low level of *luxS* expression: in the wild-type strain, *luxS* was expressed by its original promoter; however, in the luxS-C strain, *luxS* was expressed by the promoter upstream of *ermF*, which may have affected the transcription rate.

AI-2 is a side product of the bacterial activated-methyl cycle, which is a methionine-recycling pathway [15]. Inactivation of the *luxS* gene affects both AI-2 production and methionine synthesis from *S*-ribosylhomocysteine. The results of a global transcriptome analysis of an *S*. *mutans luxS* null mutant indicate that many phenotypes related to the *luxS* mutation cannot be ascribed to quorum sensing [36], and the analysis identified regulatory proteins that potentially mediate AI-2-based signaling in Gram-positive bacteria [35]. Biofilm formation by an *S*. *mutans luxS* deficient mutant was reduced compared with that of the wild type, but the effect was successfully complemented by exposure to supernatants from the wild type [25, 29]. In contrast, in the present study, biofilm formation by *C*. *ochracea* TmAI2 was not restored to wild-type levels by extrinsic AI-2 from the wild-type strain in a two compartment system, or by addition of culture supernatant of the wild-type strain. In mixed-culture, the level of biofilm formation of wild-type and TmAI2 mixed culture was significant low compared to that of the wild-type alone. The biofilm phenotype of the *S*. *sanguinis luxS*-deficient strain was not restored by AI-2 supplementation, although it was restored by transgenic *sahH*, which converts S-adenylhomocysteine to homocysteine in a single step [37]. In the present study, the addition of extrinsic AI-2 showed did not restore the biofilm-forming ability of TmAI2 to that of wild-type strain, suggesting that the contribution of AI-2 to biofilm formation is negligible. Taken together, our results suggest that a defect in the activated methyl cycle may play a major role in the attenuation of biofilm formation by *C*. *ochracea* with *luxS* inactivation.
